# The Role of GH-IGF-1 Axis and S-Klotho in Atherosclerosis Natural History, Plaque Phenotype and Vulnerability: A Narrative Review

**DOI:** 10.3390/biomedicines14040775

**Published:** 2026-03-29

**Authors:** Angela Buonpane, Salvatore Raia, Giancarlo Trimarchi, Donato Antonio Paglianiti, Fabio Casamassima, Giorgio Maria Orazi, Carlo Trani, Filippo Crea, Giovanna Liuzzo, Francesco Burzotta, Antonio Bianchi

**Affiliations:** 1 Ospedale del Cuore “G. Pasquinucci”, Via Aurelia Sud 309, 54100 Massa, Italy; angela.buonpane@santannapisa.it; 2 Scuola Superiore Sant’Anna, Institute of Life Sciences, Piazza Martiri della Libertà 33, 56127 Pisa, Italy; 3 Internal Medicine, Endocrinology and Diabetes Unit, Fondazione Policlinico Universitario A. Gemelli IRCCS (Scientific Institute for Research, Hospitalization and Healthcare), Largo Francesco Vito 1, 00168 Rome, Italy; salvatore.raia@guest.policlinicogemelli.it (S.R.); antonio.bianchi@policlinicogemelli.it (A.B.); 4 Department of Translational Medicine and Surgery, Università Cattolica del Sacro Cuore, Largo Francesco Vito 1, 00168 Rome, Italy; giorgiomaria.orazi01@icatt.it; 5 Department of Cardiovascular Sciences-CUORE, Fondazione Policlinico Universitario A. Gemelli IRCCS (Scientific Institute for Research, Hospitalization and Healthcare), Largo Francesco Vito 1, 00168 Rome, Italy; donato.paglianiti@gmail.com (D.A.P.); fabiocasamax96@gmail.com (F.C.); carlo.trani@unicatt.it (C.T.); giovanna.liuzzo@unicatt.it (G.L.); francesco.burzotta@unicatt.it (F.B.); 6 Department of Cardiovascular Sciences, Università Cattolica del Sacro Cuore, Largo Francesco Vito 1, 00168 Rome, Italy; 7 Department of Cardiovascular Sciences, Ospedale Isola Tiberina-Gemelli Isola, Via di Ponte Quattro Capi, 39, 00186 Rome, Italy; filippo.crea@fbf-isola.it

**Keywords:** atherosclerosis, plaque healing, growth hormone receptor exon 3 deletion polymorphism, insulin-like growth factor 1, S-Klotho, plaque phenotype

## Abstract

Atherosclerosis is a complex, multifactorial disease that progresses through distinct stages: initiation, progression, and complication, ultimately leading to acute coronary syndromes (ACS). Endothelial cells (ECs), vascular smooth muscle cells (VSMCs), and macrophages are central players in this process, influencing plaque stability and vulnerability. Insulin-Like Growth Factor 1 (IGF-1), soluble-Klotho (S-Klotho), and the *Growth Hormone Receptor exon 3* deletion polymorphism (GHRd3) have emerged as key modulators of vascular health, impacting these cellular components through various mechanisms. IGF-1 supports endothelial function, enhances VSMC survival and migration, and mitigates inflammation by inhibiting macrophage recruitment and activation, ultimately reducing the risk of plaque destabilization. S-Klotho, an anti-aging protein with potent anti-inflammatory and antioxidant properties, has been linked to vascular protection, with its deficiency associated with endothelial dysfunction, vascular calcification, and impaired VSMC survival. Evidence suggests that IGF-1 may enhance Klotho shedding, indicating a potential synergistic role in maintaining vascular integrity. This narrative review aims to outline the fundamental stages of atherosclerosis progression, consolidate current evidence on the roles of IGF-1 and S-Klotho in modulating key cellular components of atherosclerosis, and shed light on their potential involvement in plaque healing—an area that remains largely unexplored. By integrating established molecular mechanisms, we explore how these factors may contribute to endothelial integrity, VSMC survival, and macrophage activation and polarization, potentially shaping a more stable plaque phenotype and influencing future therapeutic strategies in cardiovascular disease.

## 1. Introduction

### 1.1. The Natural History of Atherosclerosis: From Plaque Initiation to “Plaque Healing”

#### 1.1.1. Initiation of Atherosclerosis

The pathogenesis of atherosclerosis is a complex and multifactorial process involving multiple cellular components and driven by traditional cardiovascular risk factors such as hypertension, dyslipidemia, diabetes mellitus, smoking, and obesity [1]. This pathological process unfolds through three primary phases: initiation, progression, and complication [2]. The primum movens in atherosclerotic plaque formation is endothelial injury. The healthy endothelium exhibits vasodilatory, antithrombotic, and anti-atherogenic properties. However, exposure to altered shear stress or other damaging factors (e.g., hypertension, smoking) induces endothelial dysfunction, characterized by structural and functional changes that shift the endothelium toward a prothrombotic, proatherogenic, and vasoconstrictive phenotype [2,3]. During this initial phase, the normal trilaminar structure of the arterial wall—comprising the adventitia, media, and intima—undergoes significant alterations due to the accumulation of low-density lipoproteins (LDLs) within the intima [2,3]. Once in the subintimal space, LDLs are oxidized by enzymes and reactive oxygen species (ROS) released by inflammatory cells, triggering chemokine expression by endothelial cells (ECs) and subsequently attracting leukocytes, platelets, and circulating monocytes. Monocytes infiltrate the developing plaque and, upon activation into macrophages, phagocytize oxidized LDLs (LDLox) through their scavenger receptors [2,3]. As macrophages accumulate lipids, they transform into foam cells, forming the plaque’s lipid-rich core. T lymphocytes infiltrate the subintimal space, where they regulate the activity of innate immune cells, ECs, and vascular smooth muscle cells (VSMCs). VSMCs, typically confined to the tunica media, migrate to the intima where they produce extracellular matrix components, leading to the formation of a fibrous cap, which stabilizes the plaque and prevents the thrombogenic lipid-necrotic core from interacting with circulating blood [2,3].

#### 1.1.2. Plaque Progression and Destabilization

Plaque progression is characterized by the enlargement of the lipid-necrotic core, driven by additional LDL deposition and the accumulation of apoptotic debris from macrophages and VSMCs, combined with fibrous cap degradation, ultimately promoting plaque rupture [2,3,4]. Several mechanisms may compromise plaque stability by thinning the fibrous cap. T-cell production of interferon-gamma (IFNγ) reduces the ability of VSMCs to produce extracellular matrix components, while macrophages release proteolytic enzymes that degrade interstitial collagen. Together, these factors compromise the structural integrity of the fibrous cap, increasing the likelihood of its rupture [2,3,4]. Fibrous cap rupture exposes the thrombogenic core to circulating blood components, leading to occlusive or subocclusive thrombosis, clinically manifesting as acute coronary syndrome (ACS) [4,5,6].

#### 1.1.3. Plaque Healing

Recent evidence suggests that some atherosclerotic plaques can rupture without leading to acute ischemic events, a phenomenon known as “plaque healing”. This process reflects a delicate balance between thrombogenic factors and intrinsic repair mechanisms. Plaque healing unfolds in three key phases: thrombus lysis, granulation tissue formation, and re-endothelialization [7]. The thrombus lysis phase is facilitated by endogenous fibrinolytic activity, driven by the release of tissue plasminogen activator (t-PA) and urokinase plasminogen activator (u-PA), which break down fibrin and resolve the thrombus. During the granulation tissue formation phase, VSMCs proliferate, migrate to the injured site, and synthesize extracellular matrix components, including type III collagen, to stabilize the plaque. Finally, re-endothelialization occurs, where type III collagen is gradually replaced by stronger type I collagen, and the endothelial lining is restored, completing the healing process [7]. Inflammation plays a pivotal role in plaque healing. Studies conducted on human VSMCs have shown that a T-helper1 (Th1) mediated inflammatory response, characterized by the release of IFN-gamma, reduces the ability of VSMCs to synthesize collagen. Additionally, the interaction between Th1 cells and M1 macrophages, mediated by the CD40 ligand released by Th1 cells and its receptor on macrophages, enhances the production of matrix metalloproteinases. Conversely, M2 macrophages, stimulated by IL-4 and IL-13 produced by T-helper2 (Th2) lymphocytes, appear capable of producing profibrotic factors such as fibronectin, IGF-1, and Transforming Growth Factor (TGF)-beta, thereby contributing to plaque healing [7]. This concept of plaque healing has drawn considerable attention for its potential role in repairing ruptured plaques and mitigating the risk of subsequent adverse events. However, when the healing response is insufficient or fails to fully stabilize the plaque, the risk of acute ischemic events increases. The “double-hit hypothesis” has been proposed to explain the pathogenesis of ACS. This model suggests that the combination of plaque rupture and ineffective healing serves as the two critical factors that precipitate acute ischemic events [7].

### 1.2. Plaque Phenotype and Mechanisms of Acute Coronary Syndrome

Over the past few decades, clinical and pathological studies have significantly advanced the understanding of ACS pathogenesis. While atherosclerosis alone leads to luminal narrowing and stable angina, ACS is caused by thrombus formation over unstable plaques. Plaque rupture (PR) is the most common mechanism of ACS, but other mechanisms, such as plaque erosion (PE) and eruptive calcified nodule (CN), have been identified. In this context, intravascular imaging (IVI) techniques play a key role, with Optical Coherence Tomography (OCT) standing out as the most advanced modality. Thanks to its exceptionally high spatial resolution of 10–15 μm, OCT not only enables precise characterization of atherosclerotic plaques but also allows for a clear identification of the mechanisms underlying ACS [8,9].

#### 1.2.1. Plaque Rupture

PR, accounting for approximately two-thirds of ACS, is the most prevalent mechanism underlying ACS, followed by plaque erosion and calcified nodule [6,10]. PR is characterized by a disruption of the fibrous cap, creating a cavity within the vessel wall. This disruption exposes the highly thrombogenic lipid-rich necrotic core to the bloodstream, triggering the formation of an intracoronary thrombus and the subsequent onset of ACS [6]. The mechanisms driving plaque rupture have been widely studied, making it the only ACS pathway for which a precursor lesion has been identified: the thin-cap fibroatheroma (TCFA) [4,11,12]. This type of plaque is distinguished by a large lipid core (>180°) covered by a fibrous cap thinner than 65 µm. The TCFA is considered the prototype of a “rupture-prone” plaque, serving as a key indicator of “vulnerable plaque” [11]. The concept of plaque vulnerability has been extensively investigated, and several studies have concluded that the presence of TCFA with a large lipid core (>180°) and thin fibrous cap (≤65 µm), macrophage infiltration and a small minimum lumen area (MLA) are all characteristics associated with an increased risk of adverse cardiovascular events, defining “rupture-prone” vulnerable plaque [13,14,15,16,17]. Additional features of plaque vulnerability include neoangiogenesis, characterized by the presence of microchannels [18] and cholesterol crystals (CCs) [19,20,21]. Neoangiogenesis is characterized by the presence of microvessels [18,22] growing from the adventitia into the inner layers of the vessel wall without connecting to the lumen. These microvessels allow the influx of lipid material and inflammatory cells into the plaque, promoting its destabilization. CCs are another intraplaque microstructure that may accelerate plaque progression and lead to plaque destabilization by triggering local and systemic inflammation. Their sharp, pointed shape can perforate the fibrous cap, increasing physical stress and contributing to plaque destabilization [19,20,21].

#### 1.2.2. Plaque Erosion

PE, accounting for approximately one-third of ACS cases, is characterized by endothelial loss or dysfunction without fibrous cap rupture [23,24,25]. On OCT imaging, PE is identified as the presence of thrombus or an irregular luminal surface without evidence of cap rupture. However, due to the limited resolution of OCT in detecting denudation of the endothelial monolayer, PE is typically considered a diagnosis of exclusion in vivo [8,22]. While the mechanisms underlying PR are well understood, with TCFA recognized as its precursor, the processes driving PE have only recently been explored. Unlike PR, which is associated with a specific plaque morphology, PE can occur on a variety of substrates, often involving plaques with more “stable” characteristics [23,26,27]. Previous studies, including those by Libby et al., have highlighted several factors contributing to PE, such as high endothelial shear stress, breakdown of the basement membrane, EC death, and endothelial-to-mesenchymal transition. Collectively, these processes compromise endothelial integrity, ultimately leading to intracoronary thrombosis [27].

#### 1.2.3. Eruptive Calcified Nodule

Eruptive CNs represent a distinct and less frequent mechanism of ACS, accounting for about 2–7% of culprit lesions [28]. ACN is a heavily calcified lesion protruding into the coronary artery lumen. OCT enables the differentiation of two CN subtypes: noneruptive and eruptive. Noneruptive CN is characterized by a calcified lesion protruding into the vessel lumen with a thick and intact fibrous cap without evidence of overlying thrombus. In contrast, OCT-eruptive CN is a protruding, calcified lesion, disrupting the fibrous cap, typically in the context of fibrocalcific plaques, with superimposed thrombus formation, leading to ACS [8,10,27].

### 1.3. Plaque Healing: A Double-Edged Process in Coronary Atherosclerosis

While PR, PE, and eruptive CNs are central to the pathogenesis of ACS, plaque destabilization results from a far more intricate balance between thrombogenic factors and reparative mechanisms. In the last five years, the concept of “plaque healing” has attracted significant attention, with the “double-hit theory” emerging as a framework for understanding ACS development, proposing that both plaque destabilization and an insufficient healing response are necessary to trigger an event [7]. Healed plaques, characterized by a layered structure, represent signs of previous plaque destabilization and thrombus resolution.

#### 1.3.1. Clinical Significance of “Plaque Healing”

Several studies have investigated these plaques to better understand the clinical significance of the plaque healing process. Notably, healed plaques are more frequently observed in patients with chronic coronary syndrome (CCS) [29] compared to ACS. A three-vessel OCT study by Dai et al. [30] demonstrated that layered plaques were present in three-quarters of patients with acute myocardial infarction (AMI), especially at culprit sites in STEMI cases. Furthermore, patients with layered culprit plaques also exhibited a higher number of layered non-culprit plaques, which were associated with greater lumen stenosis at both culprit and non-culprit sites compared to non-layered plaques. Several studies have highlighted the significance of layered plaques as indicators of prior plaque destabilization and markers of increased cardiovascular risk. OCT has been instrumental in identifying these plaques and understanding their morphological and clinical implications in ACS patients. A study by Russo et al., [31] analyzing ACS patients with pre-intervention OCT imaging, found that 28.4% had layered culprit plaques, which were associated with higher macrophage infiltration at non-culprit sites, suggesting widespread coronary instability rather than a localized process. Additionally, irrespective of culprit plaque phenotype, layered non-culprit plaques exhibited greater lipid content, higher prevalence of TCFA, and increased macrophage infiltration, all features that indicate increased plaque vulnerability. Patients with both layered culprit and non-culprit plaques showed the highest risk features. These findings align with those observed by Fracassi et al. [32], who investigated healed plaques in ACS patients using OCT imaging to assess their prevalence, characteristics, and clinical significance. Among 376 ACS patients, 28.7% had healed plaques, which were more common in those with diabetes, hyperlipidemia, and a history of MI. Patients with healed plaques exhibited higher systemic inflammation, as indicated by elevated high-sensitivity C-reactive protein (hs-CRP). OCT analysis revealed that PR, TCFA and macrophage accumulation were significantly more frequent in plaques with a layered phenotype, along with greater area stenosis. Although the incidence of major adverse cardiovascular events (MACE) was comparable between patients with and without layered plaques, all-cause rehospitalization was significantly higher in those with healed plaques, suggesting that while plaque healing may temporarily stabilize plaques, the presence of a layered phenotype is associated with recurrent cardiovascular complications. The combination of plaque vulnerability, local inflammation, and systemic inflammatory burden may override the protective effects of plaque healing, predisposing these plaques to future destabilization and occlusive thrombus formation.

#### 1.3.2. Plaque Healing and Plaque Vulnerability

Studies exploring the clinical significance of plaque healing have shown that healed plaques are more frequently observed in patients with CCS than in those presenting with acute coronary syndromes ACS [29]. However, these layered plaques consistently occur within a background of vulnerable plaque features and heightened systemic inflammatory activity [31,32]. This contextual association suggests that plaque healing should not be interpreted as a purely protective phenomenon. Rather, it may represent an epiphenomenon of persistent plaque vulnerability, reflecting prior episodes of subclinical destabilization and thrombus formation. In this framework, the layered morphology detected by OCT serves as a structural marker of previous plaque disruption, embedding the history of instability within the plaque architecture itself. Expanding on this concept, a recent study [33] on serial OCT imaging explored the clinical significance of newly formed layered patterns in non-culprit plaques over time. The study demonstrated that TCFA, macrophage infiltration, and thrombus were independent predictors of new layered plaque formation. Over a one-year follow-up, plaques with new layered patterns showed a progressive decrease in luminal area, a reduction in lipid content, and an increase in fibrous cap thickness. These changes indicate a paradoxical process: while plaque healing initially stabilizes the plaque, it simultaneously contributes to progressive luminal stenosis, potentially increasing the risk of future adverse cardiovascular events. These studies highlight two facets of plaque healing. On one hand, healing may play a protective role by reducing the risk of future cardiovascular events. On the other hand, it serves as a marker of coronary vulnerability, with the layered plaque structure providing evidence of prior plaque destabilization. It is important to note that plaque healing is associated with a progressive decrease in MLA, a recognized predictor of cardiovascular adverse events [13].

### 1.4. The Concept of “Pancoronary” Vulnerability and Adverse Cardiovascular Events

#### 1.4.1. Imaging-Defined Vulnerability as a Predictor of MACE

Several studies have highlighted a strong link between the vulnerable plaque phenotype and an increased incidence of MACE. Four key prospective studies have used IVI to identify high-risk patients and examine the relationship between plaque vulnerability and clinical outcomes. The ATHEROREMO-IVUS study [34] showed that TCFA lesions with a plaque burden exceeding 70% were significantly associated with a higher risk of MACE within one year. This study, which included 581 patients undergoing coronary angiography and IVUS, provided crucial evidence of this association. Further research utilizing near-infrared spectroscopy (NIRS) combined with intravascular ultrasound (IVUS) (NIRS-IVUS), including the Lipid Rich Plaque Study [35] and the ATHEROREMO-NIRS sub-study [36], demonstrated that plaques with higher lipid content were linked to poorer outcomes. Similarly, the CLIMA study [13], which evaluated 1003 patients using OCT, found that TCFA lesions with large lipid arcs (>180°) and macrophage accumulation were predictive of worse outcomes, including cardiovascular death and vessel-related myocardial infarction.

#### 1.4.2. Preventive Treatment of Vulnerable Plaques

The recognition of plaque vulnerability as a determinant of MACE has prompted investigations into whether preemptive treatment of vulnerable plaques could improve clinical outcomes. The PROSPECT ABSORB trial [37] evaluated percutaneous coronary intervention (PCI) on non-obstructive plaques in 898 myocardial infarction patients. After treating flow-limiting lesions, plaques with a burden ≥ 65% identified via IVUS-NIRS were randomized to receive bioresorbable vascular scaffolds (BVS) plus guideline-directed medical therapy (GDMT) or GDMT alone. At 25 months, BVS-treated lesions showed significantly larger minimal luminal areas, with a trend toward fewer MACE in the BVS group, though the study lacked power to confirm clinical outcomes. The PECTUS trial [38] investigated whether preemptive stenting of OCT-identified vulnerable plaques with BVS plus GDMT could reduce MACE but was halted prematurely due to the market withdrawal of the BVS Absorb device. Finally, the PREVENT trial [39], the largest study in this field, enrolled 1606 patients with ACS or CCS with non-obstructive vulnerable plaques identified via IVUS, NIRS, or OCT. This study found that preventive PCI of these plaques significantly reduced MACE compared to GDMT alone.

#### 1.4.3. Pancoronary Vulnerability and Non-Culprit Lesions

Several studies have investigated how non-culprit lesions (NCLs) can trigger future MACE, emphasizing that coronary vulnerability is not confined to a single atherosclerotic lesion but often involves the entire coronary tree. The PROSPECT (Providing Regional Observations to Study Predictors of Events in the Coronary Tree) study [14] demonstrated that in patients with ACS undergoing percutaneous coronary intervention (PCI), future MACE were equally attributable to culprit and NCLs. Most NCLs responsible for MACE were angiographically mild but exhibited high-risk features on intravascular imaging, including a plaque burden ≥ 70%, a minimal luminal area ≤ 4.0 mm^2^, or classification as TCFAs. Building on this, the PROSPECT II study [40] showed that advanced imaging with IVUS-NIRS could identify high-risk NCLs with large plaque burden and lipid content, which were strong predictors of MACE. In light of the systemic nature of atherosclerotic disease, in vivo three-vessel OCT studies have shed light on the concept of “pancoronary” vulnerability. Research by Vergallo et al. revealed that patients with plaque rupture in culprit lesions had a higher prevalence of TCFAs in NCLs, indicating a “vulnerable” atherosclerotic phenotype [41]. Another study showed that patients with non-culprit plaque rupture exhibited a pancoronary vulnerable phenotype, characterized by increased TCFA, neovascularization, and macrophage infiltration [42]. These findings extend the concept of a “vulnerable lesion” to a broader “vulnerable atherosclerotic disease.”

## 2. GH-IGF-1 Axis, IGFBPs and Cellular Mechanisms in Atherosclerosis

### 2.1. IGF-1 Receptor Signaling and Downstream Pathways

The Growth Hormone (GH)–Insulin-Like Growth Factor 1 (IGF-1) axis is central to regulating growth, metabolism, and vascular repair, involving GH, IGF-1, and insulin-like growth factor binding proteins (IGFBPs). GH stimulates hepatic production of IGF-1, a peptide that exerts systemic and local effects through endocrine, paracrine, and autocrine pathways [43]. IGF-1 operates by binding to the IGF-1 receptor (IGF-1R), a tetrameric structure composed of two extracellular α-chains and two transmembrane β-chains. The β-chains house an intracellular tyrosine kinase domain, which is essential for the receptor’s biological effects [44]. IGF-1R is expressed on various cell types, including VSMCs, ECs, and macrophages [43,44,45,46]. Binding of IGF-1 to IGF-1R induces autophosphorylation of the receptor’s intracellular tyrosine residues. Upon binding of IGF-1 to IGF-1R, autophosphorylation of the receptor occurs, triggering phosphorylation of adaptor proteins, including Shc and insulin receptor substrates (IRS-1 to IRS-4), which serve as docking proteins [44]. IGF-1R activation triggers downstream signaling pathways, primarily the PI3K/Akt and MAPK/ERK cascades, which regulate key cellular processes including proliferation, migration, differentiation, and survival [43]. The PI3K/Akt pathway promotes cell survival and enhances endothelial function, notably through increased nitric oxide (NO) production via eNOS activation [44]. In parallel, the MAPK/ERK pathway drives cellular proliferation and migration, particularly in VSMCs, contributing to vascular remodeling and repair [43,44,45,46]. Finally, two main molecular pathways regulating IGF-1 actions have been described: the PI3K/Akt pathway promoting cell survival, glucose metabolism, and NO production via eNOS activation, essential for endothelial function; and the MAPK/ERK pathway that drives cellular proliferation, differentiation, and migration, particularly in VSMCs, facilitating vascular repair and angiogenesis.

IGF-1R also forms hybrid receptors with insulin receptors, comprising one α- and one β-subunit from each receptor type. These hybrid receptors preferentially bind IGF-1 over insulin and are present in both VSMCs and ECs. However, the expression patterns differ between these cell types. In VSMCs, IGF-1R is predominantly expressed, making these cells more responsive to IGF-1 and relatively insensitive to insulin. Conversely, vascular ECs express insulin receptors alongside hybrid receptors, making them more sensitive to insulin at physiological concentrations. Insulin in ECs activates insulin receptors exclusively, rather than hybrid or IGF-1 receptors, to promote endothelial function [44].

### 2.2. Regulation by IGF Binding Proteins in Atherosclerotic Plaques

Beyond receptor signaling, IGF-1 bioavailability and activity are further regulated by IGFBPs, a family of six proteins with high-affinity binding to IGF-1 [43,47,48]. IGFBPs modulate IGF-1 bioavailability, extending its half-life in circulation while sequestering it from IGF-1R. Certain IGFBPs, such as IGFBP-3 and IGFBP-5, enhance IGF-1 activity in specific contexts, while others, like IGFBP-4, inhibit it by preventing IGF-1 from engaging IGF-1R. IGFBP-3, the most abundant IGFBP, forms a ternary complex with IGF-1 and acid-labile subunits to extend IGF-1’s half-life and transport it to target tissues [44]. IGFBP-4, an inhibitory binding protein, sequesters IGF-1 and prevents it from binding to IGF-1R, but this inhibition is reversed by PAPP-A, a metalloprotease that cleaves IGFBP-4 at sites of vascular injury [43]. In atherosclerotic plaques, IGFBPs play a dual role. For instance, IGFBP-3 has IGF-1-independent effects, promoting apoptosis via its own receptor. Plaque-derived VSMCs express elevated levels of IGFBPs, especially IGFBP-2, -3, and -4, which inhibit IGF-1-mediated survival signaling. This increased IGFBP secretion in plaque-derived VSMCs is associated with apoptosis and plaque instability, as IGF-1R activation is impaired by IGF-1 sequestration [43,45,46]. These pathways are crucial in vascular biology, particularly for VSMCs, ECs, and macrophages.

### 2.3. Effects of IGF-1 on Vascular Smooth Muscle Cells

IGF-1 exerts protective effects on VSMCs by promoting survival and reducing apoptosis through the PI3K/Akt pathway. Akt phosphorylates anti-apoptotic proteins like Bad and inhibits caspase-9, preventing apoptosis. IGF-1 also stimulates collagen synthesis, which stabilizes the fibrous cap of plaques, reducing the risk of rupture. However, plaque VSMCs in advanced plaques exhibit reduced IGF-1R expression, diminished IGF-1 binding, and impaired Akt activation, rendering them more susceptible to apoptosis [45,46,49]. Jia et al. [50] and Patel et al. [46] provide significant insights into the role of IGF-1 and IGF-1R in VSMCs and their implications in atherosclerotic plaque stability. Jia et al., [50] studied carotid endarterectomy samples from 24 patients, divided equally between symptomatic individuals (with TIA, stroke, or amaurosis fugax) and asymptomatic individuals. Their analysis revealed that VSMCs from symptomatic plaques had significantly lower IGF-1R expression compared to those from asymptomatic plaques and that this reduction in IGF-1R was associated with a higher prevalence of apoptotic VSMCs.

These findings suggest that reduced IGF-1R levels compromise VSMC survival, contributing to plaque vulnerability and increasing the risk of clinical events. Patel et al., [46] corroborated these findings in their analysis of human atherosclerotic plaques. They observed a marked reduction in IGF-1R expression in VSMCs derived from plaques, particularly those in the intima and fibrous cap regions, compared to medial VSMCs from non-diseased vessels. This loss of IGF-1R was accompanied by diminished Akt signaling, a critical pathway for anti-apoptotic and survival mechanisms mediated by IGF-1. Furthermore, their study highlighted the role of IGFBPs, which were elevated in plaque VSMCs and inhibited IGF-1’s binding to IGF-1R. This inhibitory environment further impaired the survival signaling of IGF-1, leading to increased apoptosis and plaque instability. Together, these studies emphasize that reduced IGF-1R expression and increased apoptotic VSMCs are pivotal factors in plaque vulnerability and consequent destabilization.

### 2.4. Effects of IGF-1 on Endothelial Cells

ECs rely on IGF-1 for maintaining vascular integrity. IGF-1 enhances NO production via eNOS activation, improving vasodilation and reducing oxidative stress. In diabetic or hyperlipidemic conditions, IGF-1 counteracts endothelial dysfunction by restoring insulin sensitivity and reducing ROS levels. IGF-1 also promotes endothelial repair and angiogenesis, essential for tissue regeneration [44]. Furthermore, in vitro studies have indicated that IGF-1 contributes to endothelial regeneration by enhancing circulating endothelial EPCs levels [45].

### 2.5. Effects of IGF-1 on Macrophage Modulation and Inflammatory Signaling

In macrophages, IGF-1 reduces NFκB activity, decreasing the production of pro-inflammatory cytokines like TNF-α and IL-6, inhibits macrophage recruitment and activation, thus diminishing mechanisms of plaque destabilization [44,51]. However, IGF-1 activity is inhibited in plaque regions with oxLDL, which downregulates IGF-1R and increases IGFBP-4 and IGFBP-2 production, further impairing IGF-1 signaling. Higashi et al. [51] and Okura et al. [45] provide complementary insights into the role of IGF-1 and its receptor in macrophage activity and their implications for atherosclerotic plaque stability. Higashi et al. [51], using a murine model deficient in both macrophage-specific IGF-1R and apolipoprotein E (ApoE), demonstrated that the absence of IGF-1 signaling in macrophages exacerbates atherosclerosis. In these ApoE-deficient mice, the plaques exhibited increased macrophage recruitment and proliferation, accompanied by reduced VSMC and collagen content. This led to thinner fibrous caps and features indicative of plaque vulnerability. Furthermore, macrophages lacking IGF-1R displayed a pronounced pro-inflammatory phenotype, characterized by heightened NFκB activity and elevated secretion of pro-inflammatory cytokines such as TNF-α and IL-6. Okura et al. [45] analyzed human atherosclerotic plaques and observed that decreased IGF-1 and IGF-1R expression correlated with advanced plaque stages characterized by macrophage infiltration. In regions with high macrophage density, IGF-1 and IGF-1R expression were significantly reduced, and increased VSMC apoptosis was evident, as indicated by TUNEL staining. The diminished IGF-1 signaling in macrophage-rich areas likely contributes to plaque instability by impairing VSMC survival, which is essential for maintaining fibrous cap integrity. These findings highlight the dual role of IGF-1 in suppressing macrophage-mediated inflammation while supporting VSMC viability. Together, these studies emphasize the protective role of IGF-1 and IGF-1R in promoting plaque stability through the suppression of macrophage-driven inflammation and the preservation of VSMC survival.

### 2.6. Integrative Role of IGF-1 in Vascular Homeostasis and Therapeutic Implications

**Figure 1:**
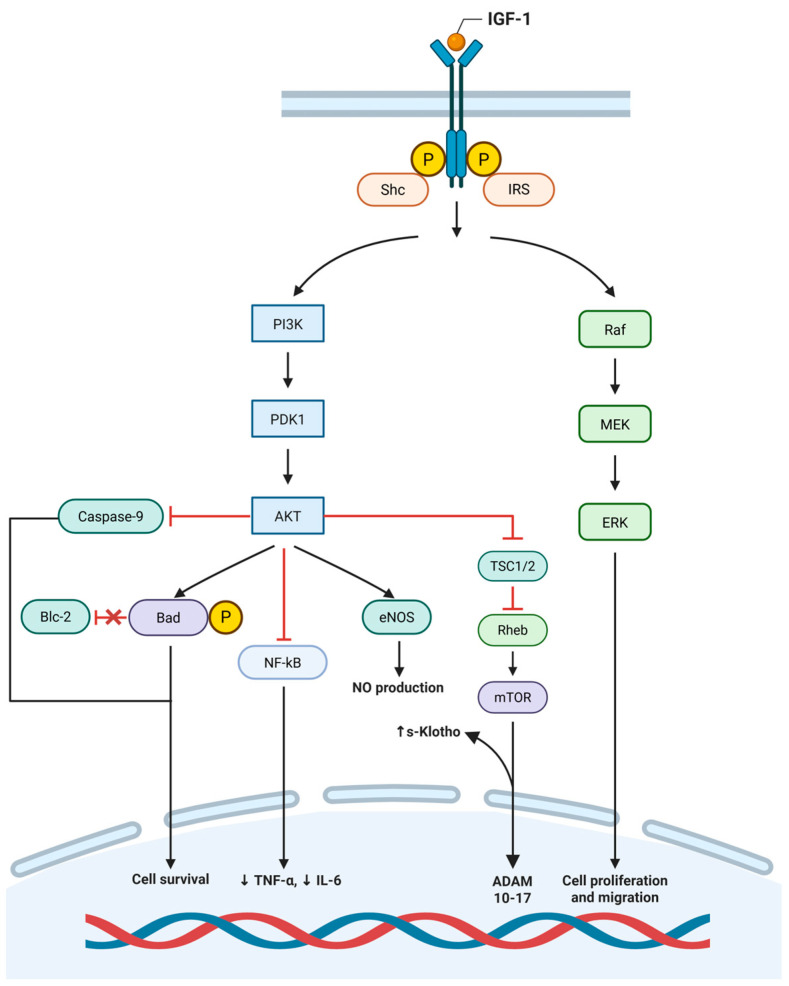
IGF-1 receptor signaling pathways and downstream mediators involved in vascular cell function.

Synthesizing all the evidence, IGF-1 appears to play a pivotal role in maintaining vascular homeostasis and promoting atherosclerotic plaque stability (Figure 1). By stimulating NO release in ECs, IGF-1 fosters a vasodilatory and anti-atherogenic environment while enhancing endothelial regeneration through increased levels of circulating EPCs. In VSMCs, IGF-1 supports fibrous cap synthesis through its mitogenic and anti-apoptotic effects, thereby stabilizing plaques, even as it may contribute to plaque progression via VSMC proliferation and migration. Furthermore, IGF-1 reduces macrophage recruitment and activation, mitigating mechanisms of plaque destabilization. Taken together, these actions position IGF-1 as a crucial factor in fostering vascular health and stabilizing atherosclerotic plaques. Targeting the GH-IGF-1 axis and modulating IGFBP activity offer therapeutic avenues for stabilizing atherosclerotic plaques. Strategies could involve enhancing IGF-1 bioavailability or using IGF-1 analogs resistant to IGFBP binding to restore anti-apoptotic signaling. Additionally, interventions aimed at reducing oxLDL levels may help normalize IGF-1 and IGF-1R expression in plaques, mitigating VSMC apoptosis and improving plaque stability.

While a substantial body of evidence supports a protective role of IGF-1 in vascular homeostasis, alternative perspectives should also be considered. Some studies have suggested that IGF-1 may contribute to atherosclerosis progression by promoting VSMC proliferation and migration, processes that are central to neointima formation and plaque growth [43,49]. In this context, the biological effects of IGF-1 appear to be strongly stage-dependent. In the early phases of atherosclerosis, characterized by intimal thickening and lesion development, IGF-1-driven VSMC proliferation and migration may contribute to neointimal expansion and plaque growth. Conversely, in advanced stages of the disease, where plaque vulnerability is determined by fibrous cap integrity and cellular apoptosis, IGF-1-mediated VSMC survival and extracellular matrix synthesis may promote plaque stabilization by enhancing fibrous cap thickness and structural resilience.

This apparent duality suggests that IGF-1 may act as a context-dependent regulator of atherosclerosis progression, exerting distinct effects according to disease stage and plaque phenotype. Future studies are warranted to better elucidate this dual role and to define the conditions under which IGF-1 signaling shifts from a protective to a potentially pro-atherogenic factor, particularly in relation to disease stage and plaque phenotype.

Binding of insulin-like growth factor-1 (IGF-1) to the IGF-1 receptor (IGF-1R) induces receptor autophosphorylation and recruitment of adaptor proteins, including insulin receptor substrate (IRS) and Src homology and collagen (Shc). These adaptor proteins couple IGF-1R activation into two major intracellular signaling pathways:

(1) PI3K/Akt pathway. Activation of phosphoinositide 3-kinase (PI3K) leads to downstream phosphorylation of phosphoinositide-dependent kinase-1 (PDK1) and subsequent activation of protein kinase B (Akt). Akt promotes cell survival through inhibition of caspase-9, phosphorylation and inactivation of the pro-apoptotic protein Bad, modulation of Bcl-2–dependent pathways, and regulation of nuclear factor-κB (NF-κB) signaling. Akt also enhances endothelial nitric oxide synthase (eNOS) activity, resulting in increased nitric oxide (NO) production. In parallel, Akt inhibits the tuberous sclerosis complex (TSC1/2), allowing activation of Ras homolog enriched in brain (Rheb) and mammalian target of rapamycin (mTOR), which regulates ADAM10/17 activity and contributes to cellular growth responses.
(2) Ras–Raf–MEK–ERK pathway. Shc-dependent activation of Ras initiates the mitogen-activated protein kinase (MAPK), promoting transcriptional programs that regulate cell proliferation and migration.

Collectively, these interconnected pathways integrate survival, inflammatory modulation, nitric oxide signaling, and proliferative responses downstream of IGF-1R activation, influencing endothelial and vascular smooth muscle cell behavior.

## 3. The Cardiovascular Protective Effects of IGF-1: Current Evidence

The protective role of IGF-1 in cardiovascular health has been extensively documented through observational studies, demonstrating its critical function in maintaining vascular integrity and mitigating cardiovascular and cerebrovascular risks. Capaldo and colleagues [52] investigated the long-term effects of childhood-onset growth hormone deficiency (GHD) on cardiovascular health. In their study of 14 GHD patients compared with matched controls, they observed significantly increased carotid artery intima-media thickness (CCI-MT), a marker of subclinical atherosclerosis. This increased CCI-MT occurred despite normal lipid profiles, indicating that GHD and the associated IGF-1 deficiency directly contribute to heightened atherosclerotic risk independent of traditional cardiovascular risk factors. Conversely, other studies suggest a strong positive association between CCI-MT and IGF-1 levels [53,54]. Laughlin et al., [55] provided further evidence of IGF-1’s importance in vascular protection through the Rancho Bernardo study. This study identified a strong association between low IGF-1 levels and increased mortality from ischemic heart disease (IHD) in older adults. Similarly, the PRIME prospective cohort study [56], which included 10,600 participants, revealed that IGF-1 levels were inversely associated with age, inflammatory markers, waist circumference, and smoking status. Moreover, individuals who later developed ACS had significantly lower baseline IGF-1 levels, and those in the highest IGF-1 quartile exhibited a 55% lower relative risk of MI.

Focusing on cerebrovascular risks, Johnsen et al. [47] have explored the relationship between IGF-1, IGFBP-3 levels, and ischemic stroke risk in a large Danish cohort. They found that low circulating IGF-1 and IGFBP-3 levels were independently associated with increased ischemic stroke risk. The study proposed that the IGF axis reduces stroke risk through anti-inflammatory effects, modulation of vascular tone, and enhanced endothelial repair, highlighting its crucial role in cerebrovascular protection. Martin and colleagues [57] supported these findings by demonstrating an inverse relationship between IGF-1 levels and the prevalence of unstable atherosclerotic plaques in older adults. In their comprehensive cross-sectional study of 310 participants aged 63–82 years, they used arterial ultrasound to assess plaque burden and stability, including measures such as arterial intima-media thickness, plaque prevalence, and plaque echogenicity. The analysis revealed that higher IGF-1 levels were associated with fewer arterial plaques and reduced echolucency, a hallmark of plaque instability.

Taken together, these studies highlight IGF-1 as a critical mediator of vascular health. Reduced IGF-1 levels, as seen in GHD and aging, are associated with increased risks of atherosclerosis, plaque instability, and ischemic events. Conversely, interventions that restore IGF-1 activity, such as GH replacement therapy, offer significant potential to improve cardiovascular outcomes by enhancing endothelial function, stabilizing plaques, and reducing inflammation.

### May Exogenous IGF-1 Act as a “Plaque Modifier”?

A previous study conducted on a porcine model of familial hypercholesterolemia tested the “plaque-modifying” role of exogenous recombinant human IGF-1. This innovative research aimed to evaluate whether IGF-1 administration could influence the progression of coronary atherosclerosis and modify plaque characteristics to enhance stability. Sukhanov et al. [58] treated hypercholesterolemic pigs with either IGF-1 or saline (control) for six months, examining the right coronary and left anterior descending arteries. The progression of plaques was monitored through IVUS at baseline (T0), three months (T3), and six months (T6), followed by post-mortem histological analyses. The findings showed that IGF-1 significantly reduced relative plaque volume and increased fibrous cap thickness, a hallmark of plaque stability. IGF-1 also decreased macrophage infiltration and necrotic core size within the plaques, shifting them toward a more stable phenotype. Molecular analyses revealed that IGF-1 reduced systemic markers of oxidative stress and inflammation, such as CXCL12 and matrix metalloproteinase 9, which are linked to plaque destabilization. Furthermore, spatial transcriptomic analyses demonstrated that IGF-1 induced significant changes in the gene expression profile of plaques. Pro-atherogenic genes like FOS/FOSB and CXCL14 were downregulated in macrophage-rich regions, supporting structural and functional stabilization of the plaques. Similarly to the study conducted by Sukhanov et al. [58], a study by Wang et al. [59] investigated the impact of IGF-1 and IGFBP-2 on atherosclerosis progression in the aorta and coronary arteries of rabbits fed a high-cholesterol diet. In this study, New Zealand white rabbits were divided into groups receiving either IGF-1, IGFBP-2, or saline for 10 weeks after being fed a 1.0% cholesterol-enriched diet for 12 weeks. The results showed that IGF-1 significantly lowered total cholesterol and LDL levels, whereas IGFBP-2 had no such effect. Moreover, IGF-1 reduced atherosclerotic lesions and macrophage accumulation within coronary artery plaques, reinforcing its potential role in plaque stabilization. In contrast, IGFBP-2 worsened atherosclerotic changes, increasing oxidative stress and inflammatory markers.

In conclusion, these studies highlight IGF-1’s potential as a “plaque-modifying” agent, reducing atherosclerotic burden and enhancing plaque stability by targeting key cellular and molecular pathways. This plaque-modifying” effect of IGF-1 closely resembles that observed with statins [60,61,62] and, more recently, with PCSK9 inhibitors [63,64,65], which have been shown to modify plaque phenotype by reducing lipid core size, increasing fibrous cap thickness, and decreasing macrophage content.

However, it is important to emphasize that the current evidence supporting IGF-1-based therapeutic strategies is predominantly derived from preclinical models, and its translation into clinical practice remains uncertain. In addition, systemic modulation of the GH–IGF-1 axis may be associated with potential metabolic and proliferative effects, raising concerns regarding safety and long-term outcomes.

Future research should therefore aim not only to better clarify the mechanisms of action of IGF-1, but also to explore more selective therapeutic approaches, such as targeted modulation of IGF-1 signaling pathways or localized delivery strategies, in order to maximize vascular benefits while minimizing systemic risks. Advanced imaging techniques, including OCT and coronary computed tomography angiography, will be essential to better characterize the impact of IGF-1 on plaque morphology and vascular health, ultimately determining its potential as a therapeutic target in cardiovascular disease.

## 4. S-Klotho: An Anti-Ageing Protein Linked to the GH-IGF1 Axis for Vascular Health

### 4.1. Biological Role of S-Klotho and Interaction with the GH–IGF-1 Axis

S-Klotho, derived from the cleavage of membrane-bound Klotho in the kidney [66], is an anti-aging protein that regulates phosphate homeostasis by acting as a cofactor for Fibroblast Growth Factor 23 (FGF-23) in the kidney, enhancing its phosphaturic effects [67,68,69]. In addition to its role in phosphate regulation, sKlotho exhibits significant anti-inflammatory and antioxidant properties [70] and several studies have shown that low sKlotho levels are associated with vascular calcification, endothelial damage, impaired neoangiogenesis, and reduced survival of VSMCs and ECs [69,71,72], highlighting its protective role in vascular health. Building on the observation that GH replacement therapy increases plasma levels of Klotho [73], several lines of evidence suggest a close correlation between IGF-1 and Klotho. Specifically, IGF-1 appears to promote klotho’s shedding via the AKT-mTOR pathway, thereby enhancing its biological effects [73].

### 4.2. Molecular Mechanisms of Vascular Protection

Low levels of sKlotho contribute to vascular damage through multiple interconnected mechanisms that extend beyond vascular calcification and arterial stiffness. The deficiency of sKlotho promotes pro-inflammatory activity, oxidative stress, impaired endothelial function, and dysfunction in VSMCs, all of which accelerate vascular aging and disease progression [71]. The pro-inflammatory effects of sKlotho deficiency are mediated by the activation of pathways such as NF-κB and the NOD-like receptor family pyrin domain containing 3 (NLRP3) inflammasome, which drive the production of pro-inflammatory cytokines. This chronic inflammatory state disrupts vascular integrity, exacerbates endothelial dysfunction, and fosters atherosclerotic plaque formation [70]. Furthermore, the absence of sKlotho’s antioxidant properties leads to increased oxidative stress, as demonstrated by its failure to counteract ROS. This oxidative damage to ECs and VSMCs initiates a vicious cycle, where oxidative stress further activates inflammatory pathways, compounding the vascular injury [69,74]. SKlotho also plays a vital role in preserving endothelial function. Its deficiency reduces NO bioavailability, a critical factor for vasodilation and vascular tone regulation. Impaired NO production results in reduced endothelial-dependent vasodilation, increased vascular tone, and heightened susceptibility to vascular injury [71]. Another crucial mechanism is the promotion of vascular calcification and arterial stiffness. sKlotho deficiency facilitates phosphate uptake by VSMCs, inducing their phenotypic shift from contractile cells to osteoblast-like cells. This transformation leads to mineral deposition within the vessel walls, increasing stiffness and reducing arterial elasticity. Simultaneously, the loss of sKlotho impairs the expression of proteins that maintain the contractile phenotype of VSMCs, further aggravating arterial stiffness and reducing the adaptive capacity of blood vessels to hemodynamic changes [71,72]. In addition, sKlotho deficiency hinders vascular repair mechanisms. Studies have shown that sKlotho supports angiogenesis and promotes the survival of ECs, both of which are critical for recovering from vascular injury [71]. These findings underscore the multifaceted role of sKlotho in vascular health. Its deficiency not only drives calcification and stiffness but also establishes a pro-inflammatory and oxidative environment, disrupts endothelial function, and impairs vascular repair. Together, these mechanisms contribute to the progression of atherosclerosis and increase the risk of cardiovascular diseases, making sKlotho a promising therapeutic target for mitigating vascular damage and promoting cardiovascular health.

### 4.3. Clinical Evidence Linking Klotho to Coronary Artery Disease

Clinical investigations have progressively linked reduced circulating Klotho levels with the presence and severity of coronary artery disease. Klotho’s systemic role in cardiovascular diseases is highlighted by Semba et al. [75], who found that reduced plasma Klotho levels are independently associated with a higher prevalence of cardiovascular disease. Similarly, Hu et al. [74] focus on the critical role of Klotho in mitigating vascular calcification in the context of chronic kidney disease (CKD). The study revealed that genetically enhanced Klotho expression in transgenic mice improved renal function, reduced hyperphosphatemia, and markedly attenuated calcification in vascular tissues. Conversely, Klotho-deficient mice exhibited exacerbated calcification. In vitro experiments further demonstrated that Klotho inhibits phosphate uptake and mineralization in VSMCs, maintaining their contractile phenotype and preventing the osteogenic transformation that contributes to calcification. These findings highlight Klotho as a critical regulator of vascular health and a potential therapeutic target for CKD-associated complications. The study by Lim et al. [69] investigates the dual role of vascular Klotho as an inhibitor of vascular calcification and a cofactor for FGF-23 signaling. It highlights that CKD induces vascular Klotho deficiency, particularly in arterial tissues, due to metabolic and inflammatory stress. This deficiency promotes vascular calcification by facilitating the osteogenic transformation of VSMCs through mechanisms such as Runx2 upregulation and serum response factor suppression. Klotho knockdown disrupts the Klotho-FGF receptor complex, rendering VSMCs resistant to the anticalcific effects of FGF-23. However, restoring vascular Klotho expression with vitamin D receptor activators reestablishes FGF-23 sensitivity and reduces calcification in VSMCs and arterial organ cultures.

### 4.4. S-Klotho and Plaque Phenotype

Given its essential role in vascular health, the relationship between Klotho levels and coronary artery disease (CAD) has become a focus of interest to better understand its potential as a biomarker and therapeutic target for atherosclerosis and related vascular conditions. Koga et al. [76] investigated the relationship between s-Klotho levels and coronary artery calcification (CAC) in 75 stable CAD patients undergoing IVUS imaging. Patients with higher serum Klotho levels showed significantly lower calcium indices (CalcIndex) in culprit lesions and across the coronary system, with an especially pronounced inverse correlation in CKD patients. Navarro-González et al. [77] analyzed 371 patients undergoing coronary angiography and 70 undergoing cardiac surgery, assessing soluble Klotho levels and vascular Klotho gene expression. Patients with significant CAD had lower serum Klotho concentrations and reduced vascular Klotho mRNA expression, while high Klotho levels were inversely associated with coronary stenosis severity, independently of traditional risk factors. Kazemi Fard et al. [74] extended the discussion by exploring Klotho’s molecular interactions in CAD. In a study involving 79 CAD patients and 78 matched healthy controls undergoing coronary angiography, CAD patients exhibited significantly lower serum Klotho levels and reduced Klotho gene expression in peripheral blood mononuclear cells, independent of traditional cardiovascular risk factors, with the degree of Klotho deficiency correlating with the severity of coronary artery stenosis. The study also examined Klotho’s interaction with the transcription factor Forkhead box protein O1 (FOXO1), involved in oxidative stress and inflammation regulation. CAD patients showed increased FOXO1 gene expression, positively correlated with Klotho expression, though FOXO1 protein levels remained unchanged, suggesting transcriptional regulation. Additionally, pro-inflammatory markers TNF-α and IL-6 were significantly elevated in CAD patients, and their levels were inversely correlated with Klotho, underscoring Klotho’s anti-inflammatory role in maintaining vascular health.

In summary, the body of evidence underscores Klotho’s multifaceted role in systemic and vascular health. Its regulatory effects on inflammation, oxidative stress, vascular calcification, and the GH-IGF-I axis establish it as a central player in maintaining cardiovascular integrity. The associations between reduced Klotho levels and CAD severity further highlight its potential as both a biomarker and therapeutic target, warranting further investigation into its clinical applications. Moreover, given the anti-inflammatory properties of S-Klotho, it is plausible to consider that this protein plays a crucial role in plaque stabilization and, consequently, in the overall clinical stability of CAD. S-Klotho is known for its protective vascular effects, including anti-oxidative, anti-senescence, and anti-inflammatory mechanisms, all of which contribute to maintaining endothelial integrity and reducing atherosclerotic progression. Inflammation is a key driver of plaque vulnerability, leading to thinning of the fibrous cap, macrophage infiltration, and increased risk of rupture, which can ultimately result in ACS. By modulating pro-inflammatory pathways, S-Klotho may reduce plaque destabilization, promote fibrous cap thickening, and inhibit macrophage-driven plaque progression.

## 5. Potential Role of the GH–IGF-1/S-Klotho Axis in Plaque Healing

Plaque healing is a dynamic process involving thrombus resolution, vascular repair, and re-endothelialization, in which inflammation, VSMC activity, and endothelial integrity play central roles. While the contribution of the GH–IGF-1/S-Klotho axis to plaque healing has not been directly demonstrated, several lines of evidence support a strong biological plausibility for its involvement. From a mechanistic perspective, IGF-1 may contribute to plaque healing by promoting VSMC survival and extracellular matrix synthesis, thereby supporting fibrous cap formation and structural stabilization of the plaque [43]. In parallel, IGF-1-mediated modulation of macrophage activity, including the attenuation of pro-inflammatory signaling pathways, may reduce local inflammatory burden and favor a microenvironment conducive to tissue repair [51]. Concurrently, S-Klotho, through its anti-inflammatory, antioxidant, and endothelial-protective properties, may enhance vascular repair processes, improve endothelial function, and facilitate re-endothelialization following plaque disruption [7]. These effects may be particularly relevant in the resolution phase of plaque healing, where restoration of endothelial integrity is critical.

Taken together, these complementary mechanisms suggest that the GH–IGF-1/S-Klotho axis may act as an integrated regulator of plaque healing, coordinating cellular survival, inflammation resolution, and vascular repair. However, although this hypothesis is supported by biological plausibility, direct mechanistic evidence remains limited, and this relationship should currently be regarded as hypothesis-generating. Further studies, particularly those integrating advanced imaging modalities such as OCT, are needed to validate this proposed link and to clarify its potential clinical implications.

To confirm this potential link, further studies will be needed, particularly those integrating advanced imaging modalities such as OCT to detect healed plaques and to assess the relationship between components of the GH–IGF-1/S-Klotho axis and the progression of plaque healing. In this context, correlating IGF-1 signaling, S-Klotho expression, and plaque phenotype may provide important insights into the temporal dynamics of vascular repair. A better understanding of these interactions could open new avenues for targeted therapeutic strategies aimed at enhancing plaque stabilization and reducing the risk of recurrent cardiovascular events. However, although the involvement of the GH–IGF-1/S-Klotho axis in plaque healing is supported by strong biological plausibility, direct mechanistic evidence remains limited, and this relationship should currently be regarded as hypothesis-generating.

## 6. The Impact of the GHR Exon 3 Polymorphism on the GH/IGF-I Axis and Clinical Implications

### 6.1. Structural and Functional Characteristics of the d3-GHR Variant

The *GHR* exists in two naturally occurring forms: the full-length (*GHR-fl*) wild-type receptor and a variant lacking exon 3 (*d3-GHR*) [78]. Observing that patients with GHD carrying the deleted form had a better response to GH therapy, it was hypothesized that *d3-GHR* is associated with enhanced intracellular signaling and greater bioavailability of IGF-1 [77,79,80] and some studies have demonstrated that this polymorphism may offer protective effects against CAD, type 2 diabetes mellitus and other metabolic disorders [78,81].

Molecular studies, such as those by Filopanti et al. [79], highlight the unique structural and functional aspects of the *d3-GHR*, which alter the receptor’s bioactivity. This deletion enhances signaling efficiency, particularly through the Stat5-dependent pathway, while maintaining receptor binding affinity and internalization. Further research, such as that by Giavoli et al. [80], has demonstrated that *d3-GHR* polymorphism affects patient responses to recombinant human GH therapy. This variant was associated with higher IGF-I normalization and alterations in lipid profiles during short-term and long-term treatment in adults with GHD. Interestingly, while the polymorphism did not influence baseline phenotypes, *d3-GHR* carriers showed distinct metabolic responses, such as improved high-density lipoprotein cholesterol levels and sustained reductions in body fat percentage. These findings align with van der Klaauw’s work [78], which observed enhanced short-term IGF-I response and lipid metabolism in *d3-GHR* carriers, although such effects diminished during extended therapy periods.

### 6.2. Cardiometabolic Protection Associated with the GHRd3 Polymorphism

The functional consequences of exon 3 deletion extend beyond receptor signaling and appear to translate into measurable differences in cardiometabolic risk. Accumulating epidemiological evidence suggests that the *d3-GHR* polymorphism may confer protection against metabolic and cardiovascular disorders [78,81]. Specifically, Strawbridge et al., [82] explored the impact of the *GHRd3* polymorphism on glucose metabolism and its association with T2DM. The study found a significantly lower frequency of *GHRd3* in individuals with T2DM compared to those with normal glucose tolerance, suggesting a protective effect of the *GHRd3* allele against T2DM, with homozygosity for *GHRd3* appearing particularly preventive. Similarly, Maitra et al. [81] explored the impact of genetic polymorphisms in the *GHR* and *GH1* genes on CAD risk within an Indian population. The research demonstrated that *GHRd3* polymorphism is significantly associated with a reduced risk of CAD. This protective effect follows a dominant inheritance model and is linked to elevated plasma high-density lipoprotein cholesterol levels, indicating potential benefits in lipid metabolism.

Finally, current evidence indicates that *GHRd3* polymorphism is associated with enhanced IGF-1 bioavailability and confers a protective effect against metabolic and cardiovascular diseases. This protection is likely influenced, at least in part, by the polymorphism’s impact on plasma IGF-1 levels. However, while the relationship between GHR polymorphism and IGF-1 levels provides a plausible explanation, it is unlikely to be the sole mechanism at play. The polymorphism may enhance intracellular signaling efficiency, alter receptor-ligand dynamics, or influence downstream pathways that regulate metabolic and vascular health. Moreover, the interplay between genetic predispositions, environmental factors, and additional molecular mechanisms could further shape the observed protective effects. These factors highlight the complexity of the relationship between the GHRd3 polymorphism and disease susceptibility.

## 7. Limitations of Experimental Models

A relevant limitation of the available evidence is that a significant proportion of the mechanistic data derives from animal models, including murine, rabbit, and porcine studies. While these models have been instrumental in elucidating molecular pathways involved in atherosclerosis, they do not fully recapitulate the complexity of human disease. Differences in lipoprotein metabolism, vascular biology, immune response, and plaque composition may limit the direct translatability of these findings to clinical settings. Moreover, key processes such as plaque rupture, erosion, and healing exhibit important species-specific differences and are not consistently reproduced in experimental models. Therefore, caution is warranted when extrapolating preclinical findings, and further validation through human studies and advanced imaging techniques is needed to confirm the clinical relevance of the GH–IGF-1 axis and S-Klotho in atherosclerosis.

## 8. Conclusions

Atherosclerosis is a complex and dynamic disease that evolves through three fundamental stages, initiation, progression, and complication [2], involving ECs, VSMCs and macrophages. These cellular elements orchestrate the intricate balance between plaque stability and vulnerability, shaping the risk of ACS. Emerging research suggests that IGF-1, S-Klotho, and GHRd3 polymorphism may significantly impact these cellular processes, influencing key mechanisms that drive vascular integrity, inflammatory response, and tissue repair. IGF-1 has been identified as a protective factor in vascular biology, with multiple studies highlighting its ability to enhance endothelial function, promote VSMC survival, and inhibit macrophage activation. The GHRd3 polymorphism, which is associated with increased IGF-1 biosynthesis, could therefore indirectly exert similar protective effects. By enhancing IGF-1 availability, this genetic variant may contribute to endothelial integrity, VSMC survival, and reduced macrophage-driven inflammation, ultimately promoting plaque stabilization and possibly facilitating plaque healing. Additionally, S-Klotho, through its anti-inflammatory properties and ability to reduce vascular calcifications, has already been shown to decrease the severity and extent of coronary artery disease. By modulating oxidative stress, inhibiting macrophage activation, and preserving VSMC function, S-Klotho may play a crucial role in vascular homeostasis and plaque phenotype and vulnerability. Further studies are needed to investigate the potential of IGF-1, GHRd3 polymorphism, and S-Klotho in promoting plaque healing. It is plausible to hypothesize that these factors play a role in this process, as they enhance VSMC survival and may contribute to macrophage polarization toward an M2 phenotype, thereby fostering a Th2-mediated inflammatory response that supports plaque repair [7].

Future studies should integrate advanced imaging techniques such as OCT, IVUS, and coronary CT angiography to assess how these factors influence plaque morphology, fibrous cap integrity, and vascular remodeling. In conclusion, IGF-1, S-Klotho, and GHR polymorphism represent promising biological regulators in atherosclerosis. Their impact on ECs, VSMCs, and macrophages places them at the center of atherosclerotic disease progression and potential therapeutic interventions. As research advances, understanding their precise role could open new avenues for personalized treatment strategies, shifting the paradigm of cardiovascular prevention from traditional risk factor modification to biologically driven approaches targeting vascular repair and plaque stabilization.

In conclusion, IGF-1, S-Klotho, and GHR polymorphism represent promising biological regulators in atherosclerosis. Their impact on ECs, VSMCs, and macrophages places them at the center of atherosclerotic disease progression and potential therapeutic interventions. As research advances, elucidating their precise and stage-dependent roles could open new avenues for personalized treatment strategies, shifting the paradigm of cardiovascular prevention from traditional risk factor modification to biologically driven approaches targeting vascular repair and plaque stabilization.

## Data Availability

No new data were created or analyzed in this study. Data sharing is not applicable to this article.

## Abbreviations

The following abbreviations are used in this manuscript:

LDL	Low-density lipoproteins
ROS	Reactive oxygen species
LDLox	oxidized LDLs
EC	Endothelial cells
VSMC	Vascular smooth muscle cells
IFNγ	Interferon-gamma
ACS	Acute coronary syndrome
TGF-beta	Transforming Growth Factor-beta
Th-1	T-helper 1
PR	Plaque rupture
PE	Plaque erosion
CN	Calcified nodule
OCT	Optical coherence tomography
TCFA	Thin-cap fibroatheroma
MLA	Minimum lumen area
CCs	Cholesterol crystals
CCS	Chronic coronary syndrome
AMI	Acute myocardial infarction
MACE	Major adverse cardiovascular events
NIRS	Near-infrared spectroscopy
IVUS	Intravascular ultrasound
BVS	Bioresorbable vascular scaffolds
PCI	Percutaneous coronary intervention
GDMT	Guideline-directed medical therapy
NCLs	Non-culprit lesions
GH	Growth Hormone
IGF-1	Insulin-Like Growth Factor 1
IGFBPs	Insulin-like growth factor binding proteins
IGF-1R	IGF-1 receptor
PI3K	Phosphoinositide 3-kinase
MAPK	Mitogen-activated protein kinase
eNOS	Nitric oxide synthase
GHD	GH deficiency
CCI-MT	Intima-media thickness
IHD	Ischemic heart disease
FGF-23	Fibroblast Growth Factor 23
NLRP3	NOD-like receptor family pyrin domain-containing 3
CKD	Chronic kidney disease
CAD	Coronary artery disease
CAC	Coronary artery calcification
FOXO1	Forkhead box protein O1
